# Factors Influencing Adjustment to Remote Work: Employees’ Initial Responses to the COVID-19 Pandemic

**DOI:** 10.3390/ijerph18136966

**Published:** 2021-06-29

**Authors:** Ward van Zoonen, Anu Sivunen, Kirsimarja Blomqvist, Thomas Olsson, Annina Ropponen, Kaisa Henttonen, Matti Vartiainen

**Affiliations:** 1 Amsterdam School of Communication Research, University of Amsterdam, Nieuwe Achtergracht 166, 1018 WV Amsterdam, The Netherlands; 2 Department of Language and Communication Studies, University of Jyväskylä, Seminaarinkatu 15, 40014 Jyväskylä, Finland; anu.e.sivunen@jyu.fi; 3 School of Business and Management, LUT University, Yliopistonkatu 34, 53850 Lappeenranta, Finland; kirsimarja.blomqvist@lut.fi; 4 Faculty of Information Technology and Communication Sciences, Tampere University, Kalevantie 4, 33100 Tampere, Finland; Thomas.olsson@tuni.fi; 5 Finnish Institute of Occupational Health, 40, 00032 Helsinki, Finland; annina.ropponen@ttl.fi; 6 UEF Business School, Kuopio Campus, University of Eastern Finland, 1627, 70211 Joensuu, Finland; kaisa.henttonen@uef.fi; 7 Department of Industrial Engineering and Management, Aalto University, Maarintie 8, 00076 Aalto, Finland; matti.vartiainen@aalto.fi

**Keywords:** work adjustment, remote work, structural factors, relational factors, contextual factors, COVID-19 pandemic

## Abstract

The COVID-19 crisis has disrupted when, where, and how employees work. Drawing on a sample of 5452 Finnish employees, this study explores the factors associated with employees’ abrupt adjustment to remote work. Specifically, this study examines *structural* factors (i.e., work independence and the clarity of job criteria), *relational* factors (i.e., interpersonal trust and social isolation), *contextual* factors of work (i.e., change in work location and perceived disruption), and *communication* dynamics (i.e., organizational communication quality and communication technology use (CTU)) as mechanisms underlying adjustment to remote work. The findings demonstrate that structural and contextual factors are important predictors of adjustment and that these relationships are moderated by communication quality and CTU. Contrary to previous research, trust in peers and supervisors does not support adjustment to remote work. We discuss the implications of these findings for practice during and beyond times of crisis.

## 1. Introduction

Even the most conservative estimates anticipate that at least 45 million jobs in the EU-27 labor market (approximately 23% of the total EU-27 employment) are directly at risk from the coronavirus disease 2019 (COVID-19) disruptions [1]. The outbreak of COVID-19 has had a drastic impact on work at a global scale [2]. Changes in when, where, and how work is completed are profound, evidenced, for instance, by widespread remote work directives [3,4,5]. The extent to which employees can adjust to remote work is crucial for individual outcomes (e.g., mental health, well-being, job satisfaction) and organizational outcomes (e.g., organizational performance). Hence, this study explores factors related to employees’ adjustment to remote work practices during the first phases of the COVID-19 pandemic. In achieving the aim of this study, we contribute to emerging research on the impact of the COVID-19 pandemic on work [3,5,6,7,8].

In line with Raghuram et al. [9], we view adjustment to remote work as an overall state of adaptation to environmental demands and conditions. Several critical indicators of adjustment to remote work have been identified, including employees’ satisfaction with remote work conditions, perceived job performance as a consequence of remote work, and the ability to balance work and non-work demands [9,10]. In contrast to previous investigations of adjustment [9], the COVID-19 crisis required organizations and their employees to abruptly change their work environments and ways of working. As such, there is a need to understand what factors are related to employees’ adjustment to remote work during this crisis [6]. Based on the theory of work adjustment [11,12] and previous work on individual adjustment in a work context [10,13] and adaptation to virtual work [9], we identify and examine a framework of environmental factors that may affect individual adjustment to remote work during the COVID-19 pandemic. Specifically, based on cross-sectional survey data, we provide empirical insights into the extent to which employees’ adjustment to remote work is associated with *structural* factors (i.e., work independence and clarity of job criteria), *relational* factors (i.e., social isolation and interpersonal trust), and *contextual* factors (i.e., perceived disruption and change in work location). Furthermore, we investigate moderating factors (i.e., organizational communication quality and communication technology use (CTU)) that potentially influence the relationships underlying employees’ adjustment to remote work. Organizational communication quality has been found to be pivotal when dealing with uncertainty and crises, including organizational responses to the COVID-19 pandemic [14]. At the same time, technological advances have been heralded for their ability to facilitate work across spatial distances and both synchronous and asynchronous collaboration [15,16,17,18]. Hence, we investigate the role of organizational communication quality and CTU in qualifying the impact of structural, relational, and contextual factors on adjustment to remote work.

Remote work settings involve temporal and spatial dispersion and depend on CTU to allow employees to interact across these boundaries [19,20,21,22,23]. Remote work is defined as “work done by an individual while at a different location than the person(s) directly supervising and/or paying for it” [24] (p. 2). In the context of the COVID-19 pandemic, this location was typically employees’ homes. Although previous studies have indicated that working from home might help employees collaborate across time zones, concentrate better than in the primary work location, and accomplish work tasks [25], it is unclear what factors impact employees’ ability to adjust to new demands of their work environment when they are mandated to work from home. We use remote work to refer to the current situation in which employees are mandated to work from home during the pandemic (i.e., the Finnish government introduced the Emergency Powers Act on 16 March, 2020, and advised all workplaces in Finland to utilize remote work if possible).

## 2. Literature Review and Hypotheses

**Figure 1:**
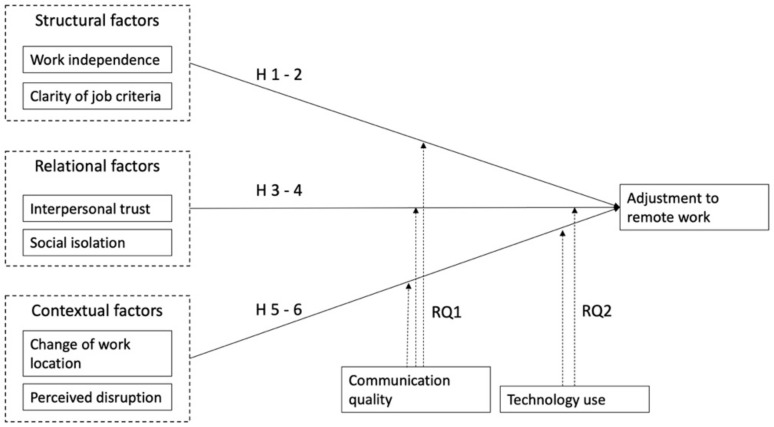
Hypothesized model.

This study takes a work adjustment perspective to examine how employees have adjusted to an abrupt transition to remote work. Adjustment to new work contexts typically involves adaptation to new environmental stimuli or demands. It has been suggested [9] that adjustment to virtual work refers to employees’ ability to adapt to virtual work modes as they transition from traditional office environments to remote work. Specifically, adjustment refers to an overall state of adaptation because a transition to remote work highlights the inherent tradeoffs involved in adjustment [9]. Several aspects are considered critical indicators of employees’ successful adaptation to a virtual work including satisfaction, commitment, productivity, and the ability to balance work and nonwork demands. Successful adaptation often requires a trade-off between these aspects. To examine the underlying factors that impact employees’ adjustment to remote work, we identified several structural, relational, and contextual factors that may impact employees’ adaptation (see Figure 1). These factors align with those proposed by Raghuram and colleagues [9], but we extend this framework by including crisis-specific concepts such as perceived disruptions and social isolation. Our conceptual model (see Figure 1) has its theoretical roots in the theory of work adjustment [11,12,26] and in the interactional model of individual adjustment [13]. It identifies several categories of factors relevant to individual adjustment, including individual, job, and organizational factors. Recently, Carillo and colleagues [6] adopted a similar approach to identify the individual, job, and organizational factors underlying telework adjustment in a crisis context. We further extend this work by exploring the role of several moderating factors, including organizational communication quality and CTU.

### 2.1. Structural Factors

Structural factors are the fundamental preconditions and organizational expectations related to a job description that may facilitate or forestall the possibilities to work remotely. Key aspects include work independence and clarity of job criteria [9]. When work can be conducted independently and the criteria for a specific job are clear, employees may be more confident in their ability to complete work remotely, facilitating better adjustment.

#### 2.1.1. Work Independence

Work independence refers to the ability of remote employees to complete tasks without having to engage in continual interaction with their coworkers [27]. Work independence can be facilitated by supporting asynchronous work, for example, allowing access to common databases through technology and ensuring that colleagues can connect with others if needed [9]. Remote employees, who must rely continually on their coworkers, thereby making them reciprocally or sequentially interdependent with others, are likely to experience time pressures, loss of control, and a decline in personal productivity [28,29]. However, independence may facilitate adjustment to remote work [9] because it allows workers to exercise more control over their behavior, for instance, when drawing boundaries between work and nonwork [30] or when enacting discipline to organize their work and apply their skills in an isolated work environment [9]. Hence, employees with greater degrees of independence are found to experience greater adjustment to a remote work context [9]. This is in line with research suggesting that individuals have an innate need for autonomy and self-determination [31,32]. Thus, we propose the following:

**Hypothesis** **1** **(H1).** *Work independence is positively associated with employees’ adjustment to remote work*.

#### 2.1.2. Clarity of Job Criteria

The clarity of job criteria means that performance assessment at work is perceived as objective, quantifiable, and transparent [9]. Clear and explicit criteria are especially beneficial to guide the performance of remote employees and develop accurate expectations among them [9,30]. This is because remote employees, compared with non-remote employees, have fewer opportunities to seek or receive informal performance feedback or clarifications from their supervisors and coworkers. Unclear evaluation criteria may lead to insecurity and uncertainty concerning work-related expectations. However, when clear and explicit evaluation criteria are in place, remote employees may be even more capable of managing themselves, which may lead to enhanced performance and satisfaction [9,30]. Additionally, clear evaluation criteria can help build mutual expectations and perceptions of procedural fairness and establish perceptions of equity among remote employees who cannot use physical behaviors to compare work outcomes [29,33]. When evaluation criteria are clearly understood, remote employees are also more likely to work on initiatives that are valued by their organization [34]. Hence, we propose the following:

**Hypothesis** **2** **(H2).** *Clarity of job criteria is positively associated with employees’ adjustment to remote work*.

### 2.2. Relational Factors

Relational factors refer to the social relationships and forms of collaboration within an organization. In the context of remote work, they relate to, for example, support and interpersonal trust [9] among employees working remotely and their supervisors and coworkers. Hence, we examine how trust can help to overcome barriers to adjusting to remote work [9]. In addition, issues of social isolation at work are especially profound during the COVID-19 pandemic; hence, we investigate how these issues may deter adjustment to remote work [35].

#### 2.2.1. Interpersonal Trust

Because remote work inherently involves physical and psychological distances, factors that create a stronger sense of relationships between coworkers may prevent physical distance from becoming psychological distance [9] and are expected to have a positive influence on employees’ ability to adjust to remote work [36]. Feelings of trust, for instance, may give remote employees greater confidence in their role within the work group or organization and facilitate further adjustment [37]. Interpersonal trust can be defined as the willingness to accept vulnerability and a positive expectation of others’ trustworthiness [38]. It has been shown to have a positive effect on workplace cooperation [39], knowledge sharing [40], and organizational commitment [41]. Trusting relations between employees [42] and employees’ trust in supervisors [43] enhance organizational change and can therefore affect how employees adjust to remote work. Trust also lowers the need for both employees and their supervisors to monitor and verify each other’s work in the virtual context [44] and is crucial to the effectiveness of virtual workers [45]. Thus, we hypothesize the following:

**Hypothesis** **3** **(H3).** *Interpersonal trust is positively associated with employees’ adjustment to remote work*.

#### 2.2.2. Social Isolation

Social isolation is related to physical and psychological distance between coworkers. Isolation can be defined as the perception of a lack of availability of support and recognition, missed opportunities for informal interactions with coworkers, and not being part of the group [35]. In other words, isolation is a state of mind or a belief that one is out of touch with others in the workplace; as such, the desire to feel socially connected is thwarted [46]. In a remote work setting, perceptions of social isolation may be exacerbated, even though it is proposed that isolation is created mainly due to the lack of availability and not just spatial distance [35]. Perceived isolation has been identified as a potential obstacle for effectiveness among remote employees [47] and may reduce job satisfaction [46]. Hence, we hypothesize the following:

**Hypothesis** **4** **(H4).** *Social isolation is negatively associated with employees’ adjustment to remote work*.

### 2.3. Contextual Factors

We extend previous models [9] of adjustment by explicitly considering the COVID-19 context within which adjustments are required. Specifically, we suggest that the severity of the change in work location—here, the discrepancy between earlier remote work experience and current remote work frequency—and the extent of broader disruptions of work routines triggered by the COVID-19 crisis impact on employees’ adjustment to the “new normal” [5,48].

#### 2.3.1. Change in Work Location

The sudden requirement to work from home has led most employees to increase the frequency with which they work from home. For some, these changes are more substantial because they either did not engage in remote work practices or did so to a very limited extent prior to the COVID-19 outbreak. In contrast, for employees who are more familiar with these work practices either because they frequently work remotely or are used to working with dispersed colleagues, these new work realities may require less adjustment. Limited connections and access in remote locations seem to be the main challenges of virtual working spaces despite recent technological advances. We examine the role of changes in work location as the discrepancy between current remote work practices and remote work practices before the COVID-19 crisis. Transitioning to a remote work location may require adjustment to the working environment, including physical, technological, and social conditions of work [16]. The intuitive hypothesis here is that larger differences indicate more substantial changes in where work is conducted, which in turn could complicate adjustment.

**Hypothesis** **5** **(H5).** *Changes in work location are negatively associated with employees’ adjustment to remote work*.

#### 2.3.2. Disruption of Work Routines

Unlearning refers to the “breakdown of routines, habits, and cognitive frameworks” [49] (p. 509). We use the term disruption to refer to an unlearning process in which routines, habits, norms, and procedures are changing [50] as a result of the COVID-19 pandemic. The adjustment required by employees depends on the level of disruption experienced by employees: greater disruption requires greater adjustment. Prior research demonstrates that environmental turbulence causes organizations and their subunits to face performance gaps, work stress, toxic work environments, and blame shifting as well as anxiety and fears [50]. Hence, we hypothesize the following:

**Hypothesis** **6** **(H6).** *Disruption of work routines is negatively associated with employees’ adjustment to remote work*.

### 2.4. Moderating Factors

In addition to structural, relational, and contextual factors, remote work is structured and shaped by communication technologies that enable employees to interact across temporal and spatial boundaries. The quality of organizational communication and the frequency of CTU in times of changing work environments and dispersed work may prove to be of particular importance for employees to adjust to new work conditions. Organizational communication quality is defined here as the informativeness, accuracy, and timeliness of communication about organizational changes during the pandemic [51].

#### 2.4.1. Organizational Communication Quality and Relational and Contextual Factors

Communication has been found to mitigate the relationship between geographical distribution and conflict [52]. Although not directly related to adjustment, these findings imply that communication might have a positive impact on conflict identification and conflict handling, and as such may optimize remote work effectiveness and satisfaction, which are key indicators of adjustment. In addition, high-quality organizational communication can be viewed as a sign of organizational support that may help employees refocus on collective goals [53] to meet their performance expectations. In the absence of a traditional office environment, the role of organizational communication may be even more profound because it may substitute in part for a lack of face-to-face interaction while facilitating the information purposes of social support networks that are normally present in organizations [54]. Finally, communication tends to clarify role expectations and enhance performance by reducing uncertainty [51]. Therefore, we ask the following question:

**RQ1:**  *Does organizational communication quality moderate the impact of structural, relational, and contextual factors underlying adjustment?*

#### 2.4.2. Communication Technology Use and Relational and Contextual Factors

Communication technologies are the enabling force behind most remote work settings, allowing workers to maintain necessary levels of connectivity to share information and coordinate work across various boundaries [55,56]. In addition, the effective use of communication technology is an important facilitator of trust in virtual teams [17,57,58]. Indeed, many organizations can be argued to have adopted some degree of virtual practices under the studied conditions, and collaboration strongly depends on the effective use of communication technology. ter Hoeven and van Zoonen [59] demonstrated that control over CTU reduces the negative consequences of spatial distance in remote work arrangements for helping behavior. Because CTU may amplify the positive association of relational factors (e.g., interpersonal trust) with adjustment while mitigating the negative impact of other relational factors (e.g., social isolation), we pose the following research question:

**RQ2:**  *Does CTU moderate the impact of relational and contextual factors underlying adjustment?*

## 3. Materials and Methods

This cross-sectional study targeted employees who had been asked to work remotely in Finland since the lockdown began in mid-March 2020. The survey started on the 26th of March 2020 and was open for responses until the 13th of April 2020. Open survey invitations were published online, and we solicited the help of several large labor unions and ministries to distribute the survey link to their members. The survey included about 100 items in total, including background questions and attention checks. The survey was administered through the XM platform Qualtrics and programmed such that all statements needed to be answered for the survey to be completed and responses to be considered for analysis. Explicit informed consent was obtained from all participants prior to the survey. Data were exported to IBM statistical software packages SPSS and AMOS for further analysis. There were no missing values as we used forced response options, respondents who failed the attention checks or dropped out were automatically excluded. Embedded data (e.g., IP addresses) and identifying information (i.e., email addresses) were assessed to guard against duplicate responses, but were not used in the analysis stage. This convenience sampling procedure resulted in a total response of 5452 Finnish employees. Employees in our sample indicated low probabilities of job loss in the near future, with 84.4% indicating that this was (highly) improbable (M = 2.07 SD = 1.43; on a 7-point scale). The average age of the respondents was 45.3 years old (SD = 10.7). Most respondents were female (N = 3747; 69%), and 1593 were male (29%). Most respondents were employed by the state or public utility (N = 3267; 60%), while 1318 respondents worked for private enterprises (24%), and others worked in nongovernmental (2%) or semigovernmental (8%) organizations. The respondents mostly worked in organizations with 50 to 249 employees (22%), 250 to 999 employees (29%), or more than 2000 employees (25%). The majority of respondents worked remotely 4 or more days per week (90.8%), 6.5% worked remotely 2 or 3 days, and 2.6% worked remotely 1 day per week or less. Furthermore, respondents indicated they worked 38.6 h per week on average (SD = 6.6), and the average reported overtime was 2.3 h per week (SD = 5.9). Approximately 14% of the respondents worked in a managerial position, their average organizational tenure was 10.9 years (SD = 10.1), most respondents were part of single (N = 1029; 19%) or two-person (N = 2152; 40%) households, and 40% had at least 1 child under the age of 18 in their household.

### 3.1. Measures

All statements were measured using seven-point response scales ranging from strongly disagree to strongly agree unless indicated otherwise. All survey items were derived from earlier studies and reviewed by the research team, but we did not conduct a pilot study. Table 1 lists all measurement items, including descriptive statistics and factor loadings.

#### 3.1.1. Dependent Variable

Adjustment to remote work was measured with a five-item scale assessing satisfaction with remote work, perceived performance as the consequence of remote work, and ability to balance work and non-work demands. The measure was adopted from Raghuram et al. [9], who used it in the context of virtual work. Adjustment refers to an overall state of adaptation because a transition to remote work highlights the inherent tradeoffs involved in adjustment [9]. For instance, research by [9] indicated that expending greater efforts to increase (or maintain) productivity may come at the expense of greater work/nonwork balance. Hence, an overall measure of adjustment may most accurately assess employees’ relative level of adaptation to environmental demands.

#### 3.1.2. Structural Factors

*Independence* was measured using four items adopted from [60]. Similar to Raghuram et al. [9], respondents were asked to indicate the degree to which their performance was dependent on working with others. *Clarity of job criteria* was measured using four items adopted from [61]. The items deal with handling problems on the job, figuring out what should be done to accomplish one’s work, and being sure of how the job needs to be done. Items are based on role conflict and role ambiguity scales proposed by House and colleagues [62].

#### 3.1.3. Relational Factors

*Interpersonal trust* was measured using four items based on [61] adopted from [9]. In line with earlier research [9], our measurement strategy focused on an overall measure of trust rather than assessing the many specific determinants of trust. Two items relate how much the individual trusts his/her supervisor and colleagues, and two items measure the extent to which the respondent perceives that his/her supervisor and colleagues trust the individual. *Social isolation* was measured using three items derived from [35]. Social isolation measures the extent to which employees feel isolated and separated from others in the workplace.

#### 3.1.4. Contextual Factors

*Change of work location* was measured by calculating the difference between remote work before the pandemic and current remote work frequency. Respondents were asked about the frequency with which they normally (before the pandemic) worked remotely, ranging from 1 (never) to 7 (6 or 7 days per week). Subsequently, we asked respondents to indicate the frequency with which they worked remotely since the moment their organization took measures related to the COVID-19 crisis. By subtracting the scores, we calculated a difference score such that a higher value indicates a larger discrepancy in remote work practices compared with normal circumstances.

*Perceived disruption* was measured by adopting eight items from [50,63]. The items address changes in work routines. Since routines are reflected in operating procedures during the performance of work, changes in plans, deadlines, and information-sharing mechanisms are indicative of an overall disruption of work. We asked employees to indicate the extent to which several activities, including project plans and deadlines, have changed since the organization took measures related to the COVID-19 crisis.

#### 3.1.5. Moderators

*Organizational communication quality* was measured using six items from Bordia et al. [64]. These items have previously been applied in the context of uncertainty during organizational change. Quality of communication was measured using items such as “the communication my organization provided adequately answered my questions about the changes.”

*Communication technology use* was measured by asking respondents to indicate the frequency with which they used various technologies to communicate with their colleagues. The communication technologies we inquired about were email, telephone, instant messaging (e.g., WhatsApp), online meetings (e.g., through Zoom or MS Teams), collaborative tools (e.g., Google Drive or Office365), enterprise social media (e.g., Yammer), and public social media (e.g., Facebook). Responses ranged from 1 (never) to 7 (hourly). The items were computed to indicate an overall score for the frequency of CTU, with higher scores indicating more frequent communication with colleagues through these technologies.

**Table 1:** Measurement items and descriptive statistics.

Measurement Items	Mean (SD)	R^2^	St. Factor Loading	Unst. Factor Loading	Se
**Adjustment to remote work [9]**					
*All in all, I am satisfied with remote work*	5.66 (1.43)	0.61	0.781	1.000	
*Remote work allows me to perform my job better than I ever could when I worked in the office*	4.39 (1.62)	0.76	0.871	1.261	0.02
*If I were given the choice to return to a traditional office environment (i.e., no longer work remotely), I would be very unlikely to do so*	3.98 (2.02)	0.43	0.657	1.184	0.02
*Since I started working remotely, I have been able to balance my job and personal life*	4.87 (1.75)	0.44	0.663	1.036	0.02
*Since I started working remotely, my productivity (e.g., sales orders, output, support) has increased*	4.39 (1.66)	0.70	0.835	1.076	0.02
**Structural Factors**					
**Independence [59]**					
*I have to obtain information and advice from colleagues to complete my work* (R) ^c^	4.15 (1.66)	0.69	0.832	1.000	
*I depend on colleagues for the completion of my work* (R)	3.85 (1.75)	0.73	0.855	1.082	0.02
*I rarely have to check in with other people to do my work*	4.72 (1.61)	0.35	0.587	0.685	0.02
*I have to work closely with other people to do my job properly* (R)	4.06 (1.82)	0.43	0.659	0.869	0.02
**Clarity of job criteria [60]**					
*I frequently don’t know how to handle problems that occur in my job* (R)	2.28 (1.30)	0.51	0.713	1.000	
*I often find that I cannot figure out what should be done to accomplish my work* (R)	1.96 (1.15)	0.76	0.869	1.073	0.02
*I am frequently confused about what I have to do on my job* (R)	1.86 (1.15)	0.80	0.894	1.100	0.02
*I am frequently unsure about how to do my work* (R)	1.94 (1.19)	0.77	0.875	1.115	0.02
**Relational Factors**					
**Interpersonal trust [9]**					
*I trust my supervisors*	5.64 (1.35)	0.60	0.773	1.000	
*My supervisors trust me*	5.84 (1.03)	0.48	0.694	0.690	0.02
*I trust my peers*	6.00 (0.86)	0.34	0.587	0.485	0.02
*My peers trust me*	5.96 (0.82)	0.29	0.541	0.422	0.02
**Social isolation [35]**					
*I am separated from my coworkers*	5.29 (1.67)	0.61	0.712	1.000	
*I often feel I am no longer close to anyone*	3.33 (1.71)	0.35	0.592	0.849	0.03
*I am isolated from others at work*	4.35 (1.82)	0.71	0.841	1.281	0.03
**Contextual Factors**					
**Remote work location ^a^**					
*How often did you normally (before the COVID-19 pandemic) work remotely (e.g., from home)?*	2.77 (1.40)	-	-	-	-
*How often have you worked at home during the COVID-19 pandemic?*	5.70 (1.05)	-	-	-	-
**Disruptions [50]**					
During the COVID-19 crisis, the following aspects of my work changed:					
*Work procedures*	4.37 (1.74)	0.39	0.624	1.000	
*Project plans*	3.67 (1.51)	0.41	0.643	0.895	0.02
*Technologies used to complete work tasks*	3.80 (1.85)	0.42	0.649	1.108	0.03
*Decision-making processes*	3.35 (1.56)	0.44	0.666	0.957	0.02
*My work tasks*	2.97 (1.66)	0.51	0.715	1.092	0.03
*The coordination of my work*	3.56 (1.66)	0.54	0.737	1.130	0.03
*The deadlines of work projects*	3.41 (1.74)	0.38	0.619	0.994	0.03
**Moderators**					
**Organizational communication quality [63]**					
*The communication my organization provided has been useful*	5.65 (1.11)	0.71	0.840	1.000	
*The communication my organization provided has adequately answered my questions about the changes*	5.50 (1.28)	0.73	0.854	1.167	0.02
*The communication my organization provided has been positive*	5.25 (1.22)	0.55	0.743	0.975	0.02
*The communication by my organization has been appropriate*	5.63 (1.11)	0.79	0.890	1.056	0.01
*The communication my organization provided has been timely*	5.25 (1.36)	0.69	0.829	1.206	0.02
*The communication my organization provided has been accurate*	5.87 (1.02)	0.58	0.760	0.826	0.01
**Communication technology use ^b^**					
Over the past two weeks, how often did you communicate about your work with colleagues using					
*Phone calls*	3.08 (1.37)	-	-	-	-
*E-mails*	4.79 (0.94)	-	-	-	-
*Online meetings (e.g., Skype, MS Teams, Zoom)*	4.27 (1.05)	-	-	-	-
*Text or instant messaging (e.g., WhatsApp, Messenger)*	3.06 (1.64)	-	-	-	-
*Collaboration tools (e.g., Office 365, Google Drive)*	2.81 (1.75)	-	-	-	-
*Enterprise social media (e.g., Yammer, Happeo)*	1.91 (1.45)	-	-	-	-
*Public social media (e.g., Facebook, Twitter)*	1.58 (1.15)	-	-	-	-

Notes: ^a^ Change was calculated as a difference score between two observed variables and therefore not included in the CFA; ^b^ a sum score indicating the average frequency of communication technology use was calculated and therefore not included in the CFA. ^c^ (R) indicates that items were reverse coded.

## 4. Results

### 4.1. Measurement Model

A confirmatory factor analysis (in AMOS) was used to examine the hypothesized factor structure and investigate the validity of our measurement model. Subsequently, we examined common method variance using a common latent factor approach.

The model demonstrated good model fit: χ^2^ (469) = 4432.49; CFI = 0.96; TLI = 0.95; SRMR = 0.04; PClose 1.000; and RMSEA = 0.039 (CI: 0.038, 0.040). Following recommendations and threshold values reported by Hair et al. [65], the model demonstrated convergent and discriminant validity of the measures in our model (see Table 1). The average variance extracted (AVE) ranged between 0.43 and 0.71. Discriminant validity was examined through the maximum shared variance (MSV), which ranged between 0.07 and 0.37 for the constructs in our model and is smaller than the AVE values. Additionally, the square root of the AVE was greater than the inter-construct correlations. Inspection of the model parameters indicated the absence of cross-loadings, overall suggesting good discriminant validity. Reliability was examined through the composite reliabilities (CR) and the maximum reliability (H), which ranged between 0.75 and 0.93 and between 0.77 and 0.93, respectively.

Second, we examined common method variance using a common latent factor approach. Squared regression estimates indicated that common method variance was 3.6%, indicating that common method variance is not a substantial concern in our data. Curve estimations for all relationships in our model indicated that these relationships were sufficiently linear. Finally, the correlation between interpersonal trust and communication quality was relatively high (0.61; see Table 2). Hence, we inspected collinearity statistics (i.e., the variance inflation factor, VIF) for all independent variables and discovered no problems with multicollinearity. In sum, these results justify further inspection of the structural model.

### 4.2. Controls

We considered several potentially confounding factors in our analysis. Specifically, we controlled for gender, age, working hours per week, managerial position, organizational tenure, and job security. Gender significantly predicted adjustment (B = 0.091, *p* = 0.009), suggesting that female respondents were better able to adjust to remote work. Gender did not affect any of the hypothesized relationships in the model. Age did not affect adjustment to remote work (B = 0.000, *p* = 0.766). Similarly, the number of work hours per week did not significantly affect adjustment to remote work (B = −0.001, *p* = 0.691). However, the results indicated that managerial positions had a significant and negative relationship with adjustment (B = −0.176, *p* < 0.001), suggesting that individuals in managerial positions seem to have more difficulties adjusting to remote work. Finally, organizational tenure (B = −0.001, *p* = 0.202) and job security (B = 0.012, *p* = 0.274) did not affect adjustment to remote work or any of the relationships in our model. In sum, all hypothesized relationships remained unaffected when these variables were included. Hence, these variables were excluded from the final model for reasons of parsimony.

### 4.3. Hypotheses Testing

The hypothesized model was examined using path modeling in AMOS by estimating regression coefficients between the structural, relational, and contextual factors on adjustment to work. Table 3 provides the standardized and unstandardized regression results for the full model.

*Structural factors*. Hypothesis 1 assumes that work independence is positively related to adjustment to remote work. The results demonstrate a significant positive relationship (B = 0.168 (0.143; 0.192), *p* = 0.001). Hence, hypothesis 1 is supported. In addition, hypothesis 2 reflects the assumption that clarity of job criteria makes it easier for employees to adapt to remote work. The findings demonstrate a significant positive relationship between the clarity of job criteria and adjustment to remote work (B = 0.174 (0.136; 0.211), *p* = 0.001). Hence, hypothesis 2 is also supported. Overall, these results provide strong support that the structural factors of an employee’s job have an important influence on the employee’s adjustment to remote work.

*Relational factors*. Hypothesis 3 posits that interpersonal trust is positively related to employees’ adjustment to remote work. The results demonstrate a significant negative relationship between trust and adjustment (B = −0.069 (−0.117; −0.021), *p* = 0.006). Hence, contrary to our expectations, trust between coworkers and supervisors does not increase adjustment to remote work but rather decreases it. A possible explanation could be that employees who exhibit lower levels of trust in their peers and supervisors rather work (alone) remotely, as this gives them more autonomy from people they do not trust and, therefore, they are less frequently confronted with such relationships. However, as the relationship is in the opposite direction than the one we hypothesized, we do not find support for hypothesis 3. Hypothesis 4 suggests that social isolation is negatively related to adjustment to remote work. The results demonstrate a significant negative relationship between perceived social isolation and adjustment to remote work (B = −0.178 (−0.202; −0.152), *p* = 0.001), providing support for Hypothesis 4.

*Contextual factors*. Hypothesis 5 reflects the rationale that the relative change in work locations influences employees’ adjustment to remote work. The results show that a change in work location is negatively related to adjustment to remote work (B = −0.209 (−0.234; −0.186), *p* = 0.001). This result suggests that a larger change in work location (e.g., a change in remote work from half a day per week to five days per week versus a change in remote work from two days per week normally to five days per week currently) reduces employees’ adjustment to remote work. Hence, hypothesis 5 is supported. This implies that employees who were already used to working remotely before the pandemic adjusted better to the new situation. Hypothesis 6 suggests that perceived disruption is negatively related to adjustment to remote work. The findings demonstrate a significant negative relationship (B = −0.122 (−0.153; −0.093), *p* = 0.001), providing support for hypothesis 6. The more work practices changed during the pandemic, the less employees were able to adjust.

### 4.4. Moderations

Before discussing the interactions, it should be noted that both moderators, organizational communication quality (B = 0.114 [0.075; 0.152], *p* = 0.001) and the frequency of CTU (B = 0.103 [0.059; 0.152], *p* = 0.001), are significantly and positively related to adjustment. Note that all variables that comprise product terms were mean centered prior to testing the interactions. For all interactions, we inspected the values of the interactions effect at different values of the moderator using the Johnson–Neyman technique. When the interaction reported was not significant at all values of the moderator, we reported the value of the moderator at which the interaction becomes significant. To facilitate interpretation, the mean-centered values are also reported as actual (raw) values.

*Organizational communication quality*. There was no significant interaction effect between organizational communication quality and trust (B = 0.002 (−0.039; 0.041), *p* = 0.995), social isolation (B = 0.019 (−0.007; 0.043), *p* = 0.149), and disruption (B = 0.007 (−0.025; 0.036), *p* = 0.722) on employees’ adjustment to remote work. There was a significant interaction between organizational communication quality and change in work location (B = −0.045 (−0.070; −0.021), *p* = 0.001) on adjustment to remote work. This result suggests that at low levels of organizational communication quality starting at −4.11 (i.e., 1.42 in raw values), perceived change in work location negatively impacts adjustment to remote work. Organizational communication quality has a limited impact in mitigating this negative relationship. Finally, we did not find significant interactions between organizational communication quality and clarity of job criteria on adjustment (B = −0.010 (−0.044; 0.024), *p* = 0.620), nor did we find an interaction between communication quality and job independence on adjustment (B = 0.014 (−0.012; 0.039), *p* = 0.263).

*Communication technology use*. There were no significant interactions between social isolation and CTU (B = 0.009 (−0.007; 0.043), *p* = 0.149). CTU was found to moderate the negative relationship between trust and adjustment to remote work (B = −0.107 (−0.168; −0.046), *p* = 0.002). The result indicates that trust negatively affects adjustment to work when the mean-centered value of CTU is below 0.562 (i.e., 3.63 in raw values). This suggests that when CTU is low, trust stifles adjustment to remote work, but when CTU is high (above 0.562, approximately 22% of the responses), there is no significant negative effect of trust on adjustment to remote work. Arguably, frequent CTU is important when trust is low, to mitigate a decline in employees’ commitment, satisfaction, and productivity. However, CTU is also important for adjustment when trust is high as a lack of communication may be more detrimental to adjustment in high-trusting environments. For instance, trust may be an indicator of high-quality relationships, missing out on such relationships in remote work settings may be detrimental to several aspects of adjustment, increasing the frequency of CTU to communicate and collaborate with trusted peers and supervisors might reduce the negative impact on employees’ adjustment.

In addition, CTU was found to moderate the negative relationship between change in work location and adjustment (B = −0.031 (−0.065; −0.001), *p* = 0.045). The findings suggest that change is negatively related to adjustment at all levels of CTU. However, smaller levels of change and a higher frequency of CTU yield the highest levels of adjustment. At one standard deviation below the mean (−1.92) of change, adjustment is higher (5.33) when CTU is one standard deviation above the mean (0.786) rather than below the mean (−0.786), in which case adjustment is 5.07. Finally, CTU moderates the relationship between disruption and adjustment to remote work (B = 0.050 (0.013; 0.085), *p* = 0.006). The confidence interval of the slope indicates that disruption has a negative impact on adjustment to remote work at all levels of CTU. However, it should be noted that higher frequencies of CTU allow employees to adjust better to remote work when disruption is high than when the frequency of CTU in these situations is low.

## 5. Discussion

The findings of this cross-sectional study during the early phase of the COVID-19 outbreak in 2020 in Finland indicate that structural factors (i.e., high work independence and clarity of job criteria) make it easier for employees to adjust to remote work settings. In turn, relational factors (i.e., interpersonal trust and isolation) are negatively related to adjustment. Contrary to our expectations and to the earlier findings by [9], interpersonal trust was negatively associated with adjustment to remote work. Arguably, trust serves as a proxy for important interpersonal functions, such as socialization and support; when such cues are missing, employees may feel less satisfied and effective and may therefore experience lower levels of adjustment to remote work. Our findings also show that feelings of social isolation decrease adjustment to remote work, providing further evidence that the social dynamics of work present a key barrier in adjustment during the COVID-19 pandemic. Furthermore, the results indicate that greater discrepancy between the amount of current and “normal” remote work and greater disruption in work practices (i.e., contextual factors) both decrease adjustment. These results imply that beyond smaller changes in work location, greater experience with remote work seems to enhance adjustment to remote work. Finally, the findings demonstrate a relatively small positive impact of organizational communication quality and CTU in adapting to increased remote work. Organizational communication quality does not mitigate the negative impacts of relational factors on adjustment (i.e., interpersonal trust and feelings of isolation) or facilitate the relationship between disruption of work practices and adjustment. However, more frequent use of various communication technologies with colleagues seems to mitigate the negative relationship between trust and adjustment, probably by reviving social relations. More research is needed to study the potential buffering effects of communication technology use and remote work adjustment.

### 5.1. Theoretical Implications

The findings have several theoretical implications. First, based on the theory of work adjustment [12] and the interactional model of individual adjustment [13], this study identified several crisis-specific environmental factors [6] in addition to “traditional” environmental factors proposed by [9] that may underlie employees’ adjustment to remote work. For instance, we demonstrated that isolation is a relevant predictor that could be conceptualized as a relational factor underlying adjustment. In addition, we conceptualized contextual factors that include crisis-specific indicators such as changes in work location and perceived disruptions that impact adjustment. Finally, this study further considered how communication quality and CTU may mitigate some of the challenges in adapting to remote work. Ultimately, the study contributes to the literature on adjustment by identifying how employees’ ability to adjust to abrupt transitions to remote work has been affected by various relevant factors of the work environment. Specifically, this study contributes to person–environment theories and the theory of work adjustment by identifying how the work environment may enable or constrain employees’ ability to adapt. In other words, the work environment has reinforcement capabilities that can satisfy a person’s needs (in this case, adjustment) [12].

Second, work independence and clarity of job criteria were positively and significantly related to work adjustment. This suggests that employees who know what is expected from them and can complete their tasks without others adjust better to working remotely. Hence, with regard to the structural factors underlying remote work, we were able to replicate the findings presented by [9] in the context of virtual work and add that these factors operate in similar ways across organizational settings during a global health pandemic characterized by abrupt lockdowns and en masse remote work directives. Furthermore, the findings align with [66], who find that telecommuters with higher autonomy report greater job satisfaction relative to those with less autonomy. Our findings are in line with previous literature linking performance management and goal-setting theory in co-located work settings. It has been established that specific goals can enhance motivation and performance by leading people to focus their attention on specific objectives [67], facilitate their attempts to achieve these objectives [68], persist in the face of setbacks [69], and invent new strategies to better deal with complex challenges related to goal attainment [70].

More broadly, the finding that work independence and clarity of job criteria are positively related to adjustment also signals a potentially important tension in remote work designs. While some level of independence and clarity at the individual level is desirable for job satisfaction, effectiveness, and performance in remote settings, modern work tasks require some level of interdependency, and employees may desire feedback, socialization and relatedness with peers. For example, in self-determination theory, relatedness is considered a basic human need that consists of interacting with, being connected to, and experiencing caring for others [31]. Recent studies on remote work [69] and global work [54] demonstrate the importance of considering both the job characteristics (e.g., complexity and problem solving) and the social characteristics (e.g., social support and interdependence) of work. Research [54,55] indicates that as workers are afforded more autonomy and work becomes more unpredictable and volatile, employees need to adapt to contend with the demands of their work environment, including relational demands. This means that employees are active agents crafting their own jobs rather than passive recipients of work characteristics. This perspective aligns well with the findings of this study, which refer to agentic processes—here, independence and clarity, which empower employees to meet the demands of remote work and to adjust.

Third, with regard to the relational factors, our findings do not support the hypothesis of a positive relationship between interpersonal trust and adjustment to remote work. The negative relationship between trust and remote work adjustment is a counterintuitive finding that contradicts most of the past research on the relationship between trust and remote work [9,71]. The findings indicate that higher levels of interpersonal trust decrease individual’s adjustment to remote work. Alternatively, the reverse is also true as employees who report low levels of trust seem to adjust better to remote work. This result can be understood from an “out of sight out of mind” perspective, suggesting that some employees may benefit from being separated from colleagues or supervisors they do not trust, or even distrust. Indeed, in the context of this pandemic, scholars have chronicled that the transition to remote work may have benefits for employees as they might be less exposed to toxic workplace relationships, or relieved from bullying colleagues [5]. Our findings align with such insights. In addition, trust among colleagues and in supervisors could be an indication of a valued interpersonal relationship. Having to miss such a relationship may reduce one’s satisfaction with their job and make it more difficult to maintain productivity levels or overall job performance—all indicators of adjustment. Hence, employees who report high levels of trust in coworkers and supervisors and who feel trusted by them may be less satisfied with remote work, feel less effective, and feel less adjusted. Employees may even want to return to the office as soon as possible to reconnect with their colleagues because the gratification of social needs is arguably satisfied through recurring physical interactions with colleagues. Furthermore, trust built in the physical context may not have transferred to the technology-mediated interactions of remote work environments yet, and if the respondents see limited opportunities to do so, this could lead to a less gratifying remote work experience.

Finally, the notion that greater disruption requires greater adaptation certainly rings true for most individuals. The findings suggest that employees who experience greater disruption appear to face more difficulties adjusting to the work setting. Hence, it seems that disruption may indeed require adaptation, but the negative relationship suggests that employees have difficulty making the required adjustments, arguably because adaptation in these cases requires employees to learn new skills and competencies to deal with environmental demands. Hence, the findings demonstrate that employees’ adjustment to abrupt remote work transitions is complicated by the perceived “strength” of the disruption. Overall, the findings suggest that factors underlying agentic processes (i.e., independence and clarity of job criteria) facilitate adaptation and the reappraisal of event outcomes, while relational factors—trust and isolation—operate as barriers to adjustment. This phenomenon calls for further research into the managerial and sociopsychological processes that help to understand the relationship between disruptive events and organizational outcomes.

### 5.2. Practical and Managerial Implication

This study investigated the antecedents of adjustment to remote work. Some factors are directly within the scope of organizational control (e.g., structural factors), while others may be more difficult to influence directly (e.g., contextual factors). However, the results provide important and actionable implications for organizations. First, our results indicate that employees who report higher levels of independence and clarity of job instructions are better able to adjust to remote work. In these circumstances, organizations could provide clearer objectives and goals (decreasing ambiguity) and minimize interdependencies between organizational members by designing and allocating autonomous jobs and tasks where possible. In doing so, organizations facilitate agentic processes of individual employees, improving their adjustment to these settings. In addition, organizations need to ensure that there are adequate resources for employees to conduct work independently while maintaining interdependencies at the collective level. For instance, our findings show that CTU in particular, as well as organizational communication quality, may bolster adjustment to remote work.

Managing relational factors deserves slightly more thought because higher levels of trust reduce adjustment, but isolation also reduces adjustment. Social isolation can be reduced in various ways, such as through synchronous video meetings and informal communication. For example, virtual coffee breaks may help employees feel connected to their coworkers and may lead to less isolation in the workplace. In addition, these initiatives might be important in the context of trust. We found a negative relationship with adjustment; however, we argue that trust in this case signals the absence of important interpersonal cues in the physical workplace. Hence, facilitating interpersonal mechanisms of socialization and support might mitigate the negative impact of trust on adjustment. Furthermore, to facilitate greater adjustment in times where resources could be particularly scarce and feelings of isolation particularly high, scholars have suggested that teleconsultations and informal online support groups could help people stay connected [72,73,74].

In addition, the findings demonstrate that greater change in work location and greater perceived disruption hamper adjustment to remote work. This is important because it signals that organizations and managers should be attentive, especially to employees whose work processes require the greatest adaptation. Our findings show that employees with more experience in remote work adapt to new situations better because they have already learned some practices and competencies needed in remote work. This implies the importance of training. Organizational support for work–home issues significantly improves well-being [75] and may aid adjustment. For instance, organizations may support their employees through lower workloads or other job demands, giving them greater opportunities to adjust. Additionally, for employees working on vital processes, organizations could have different approaches based on the extent to which the work routines of individuals or groups are disrupted. For instance, these workers could be given priority to use workplace facilities.

### 5.3. Limitations and Future Research

Several limitations need to be acknowledged. First, this study relies on cross-sectional survey data obtained through a convenience sampling method. This method presents two limitations. First, the data do not permit any causal inferences and do not permit us to track how changes in, for instance, perceived disruptions and continued adjustment to the work environment develop over time. Second, the sampling method resulted in a relatively homogeneous group of employees, limiting the generalizability of the findings. The nonrandom sampling technique as well as the sample and population information do not provide sufficient auxiliary information to correct survey responses using weight adjustments. The sample consisted predominantly of Finnish civil servants (60%), many of whom engaged in what could best be described as knowledge work. In addition, we surveyed respondents who had the available means to participate in the study (e.g., stable Internet connection and time). Furthermore, the relatively stable work context of these employees and the Finnish socioeconomic system may be fertile ground for the adjustment of employees, which may not be the case in other types of occupations [5], other socioeconomic systems, or countries that were (at least at the time of the study) more strongly affected by the pandemic (e.g., France, Italy, and Spain) [6]. Future research is needed to demonstrate the generalizability of our findings across a broader range of occupations, countries, and socioeconomic systems.

Second, some of the effect sizes are relatively small, which raises questions about the predictive validity of the model. To substantiate these findings, future research is needed to confirm these results over time with various occupational groups in different socioeconomic systems. In addition, although the measures were adopted from previous studies, not all measures have been validated and we do not have pre-pandemic benchmark indices for our population, limiting our ability to draw a conclusion about the relative changes. In addition, responses were collected within the first month of the outbreak. Although this timeframe is considered appropriate to investigate adjustment processes, these processes are also likely to continue as the pandemic evolved. In addition, people now have had more time to adjust and find ways to meet challenges and demands associated with teleworking. Hence, it would be worthwhile to follow up on this study as the relative newness of the pandemic and associated teleworking has decreased. Finally, our findings suggest that employees seem to adjust well to remote work. This aligns with studies that conclude that the general attitude toward working from home seems positive [76]. However, this study was conducted in the early stages of the pandemic; therefore, limited assumptions can be made about the long-term implications. In addition, future research may probe more deeply into different aspects that are central to adjustment to remote work, such as those related to work–life dynamics. For instance, now is the opportune time to study whether childless and single employees face increased expectations and work responsibilities, and how these demands may interfere with non-work demands [3].

## 6. Conclusions

The findings presented in this study provide important insights into the factors that are consequential to employees’ adjustment to remote work. These findings contribute to our understanding of how the COVID-19 pandemic has impacted work. This is important because the current crisis is far from over [74,77], future pandemics are increasingly likely [74], and other disruptive events, such as economic downturn, natural disasters, activism, and war, may require continuous adjustment from employees and organizations. Our findings contribute to an understanding of how employees adjust to (abrupt) changes in their work environment by identifying and demonstrating the interplay between various environmental and contextual factors.

## Figures and Tables

**Table 2:** Correlation Matrix of variables with validity statistics.

Variable	M (SD)	CR	AVE	MSV	MaxR(H)	1	2	3	4	5	6	7	8	9
1. Independence	4.20 (1.40)	0.83	0.55	0.07	0.86	**0.74**								
2. Clarity of job criteria	5.99 (1.05)	0.91	0.71	0.10	0.92	−0.27	**0.84**							
3. Interpersonal trust	5.86 (0.82)	0.75	0.43	0.37	0.77	0.06	0.32	**0.66**						
4. Social isolation	4.32 (1.41)	0.76	0.52	0.09	0.80	0.03	−0.25	−0.10	**0.72**					
5. Disruption	3.59 (1.21)	0.85	0.44	0.08	0.85	0.13	−0.28	−0.12	0.15	**0.67**				
6. Change ^a^	3.92 (1.53)	−	−	−	−	0.14	−0.11	0.01	0.15	0.18	**–**			
7. Communication quality	5.53 (1.01)	0.93	0.67	0.37	0.93	0.05	*0.22*	0.61	−0.13	−0.11	−0.01	**0.82**		
8. Technology use ^b^	3.07 (0.75)	−	−	−	−	0.27	0.10	0.09	0.13	0.12	0.07	0.11	**−**	
9. Adjustment	4.66 (1.34)	0.88	0.59	0.10	0.90	−0.25	0.32	0.06	−0.30	−0.26	−0.32	0.15	0.06	**0.77**

Notes: CR = composite reliability; AVE = average variance extracted; MSV = maximum shared variance; MaxR(H) = maximum reliability. Square root of the AVE is reported on the diagonal. ^a^ Change was calculated as a difference score between two observed variables and therefore not included in the CFA; ^b^ a sum score indicating the average frequency of communication technology use was calculated and therefore not included in the CFA. Technology use is treated as the index score, where higher scores mean higher general technology use. All correlations equal to or above 0.03 are significant at *p* < 0.05.

**Table 3:** Parameter estimates of path model.

					Bootstrapping BC 95% CI		
		B	SE	Beta	Lower	Upper	*p*
**Hypotheses**							
*H1*	Independence → Adjustment	0.168	0.012	0.175	0.143	0.192	0.001
*H2*	Clarity of job criteria → Adjustment	0.174	0.017	0.136	0.136	0.211	0.001
*H3*	Interpersonal trust → Adjustment	−0.069	0.023	−0.042	−0.117	−0.021	0.006
*H4*	Social isolation → Adjustment	−0.178	0.012	−0.188	−0.202	−0.152	0.001
*H5*	Remote work transition → Adjustment	−0.209	0.011	−0.239	−0.234	−0.186	0.001
*H6*	Perceived disruption → Adjustment	−0.122	0.014	−0.110	−0.153	−0.093	0.001
**RQ1: Communication quality × Relational and contextual factors**							
*RQ1*	Communication quality × Trust → Adjustment	0.002	0.015	0.002	−0.039	0.041	0.995
	Communication quality × Isolation → Adjustment	0.019	0.010	0.023	−0.007	0.043	0.149
	Communication quality × Change in location → Adjustment	−0.045	0.010	−0.057	−0.070	−0.021	0.001
	Communication quality × Disruption → Adjustment	0.007	0.130	0.007	−0.025	0.036	0.722
	Communication quality × Independence → Adjustment	0.016	0.011	0.014	−0.012	0.039	0.263
	Communication quality × Clarity of job criteria → adjustment	−0.009	0.015	−0.010	−0.044	0.024	0.620
**RQ2: Communication technology use × Relational and contextual factors**							
*RQ2*	Technology use × Trust → Adjustment	−0.107	0.024	−0.053	−0.168	−0.046	0.002
	Technology use × Isolation → Adjustment	0.009	0.015	0.007	−0.007	0.043	0.149
	Technology use × Change in location → Adjustment	−0.031	0.014	−0.028	−0.065	−0.001	0.045
	Technology use × Disruption → Adjustment	0.050	0.017	0.035	0.013	0.085	0.006

Notes: Bootstrapping is a technique from which the sampling distribution of statistic is estimated by taking repeated samples from the dataset. Bootstrapping was used to obtain model estimates. BC95% CI indicate the bias-corrected 95% confidence interval of the beta coefficient.

## Data Availability

The data presented in this study are not publicly available. Survey respondents were assured raw data would remain confidential and would not be shared.
